# Individualised treatment targets in patients with type-2 diabetes and hypertension

**DOI:** 10.1186/s12933-018-0661-8

**Published:** 2018-01-22

**Authors:** Roland E. Schmieder, Diethelm Tschöpe, Cornelia Koch, Taoufik Ouarrak, Anselm K. Gitt, for the DIALOGUE study group, Sibel Avsar, Peter Bramlage, Eva Duetting, Anselm K. Gitt, Cornelia Koch, Alfons Müller, Alexander Neumer, Taoufik Ouarrak, Roland E. Schmieder, Steffen Schneider, Diethelm Tschöpe

**Affiliations:** 1 Medizinische Klinik 4, Nephrologie und Hypertensiologie, Universitätsklinikum Erlangen, Ulmenweg 18, 91054 Erlangen, Germany; 2 Diabeteszentrum am Herz- und Diabeteszentrum Nordrhein-Westfalen, Ruhr Universität Bochum, Bad Oeynhausen, Germany; 3 Novartis Pharma GmbH, Nürnberg, Germany; 4 Institut für Herzinfarktforschung, Ludwigshafen, Germany; 5 Medizinische Klinik B, Herzzentrum Ludwigshafen, Ludwigshafen, Germany

**Keywords:** Diabetes, Hypertension, Glucose, HbA1c, Target, Hyperglycaemia, Individualised

## Abstract

**Aim:**

Patients with type-2 diabetes mellitus (T2DM) are at high risk of cardiovascular events, accentuated in the presence of hypertension. At present, it is unclear to what extent the guidelines for the management of T2DM, advocating reduction in HbA1c levels to below target levels, are being adhered to in clinical practice.

**Methods:**

DIALOGUE was a prospective, observational, non‐interventional registry performed across multiple centres in Germany. Patients aged 18 years or older who had T2DM and hypertension for whom the treating physician considered blood glucose lowering medication as inadequate and/or not safe/tolerable and chose to add a further oral drug or switch drug treatment were included. Patients were assigned a treatment target HbA1c value (≤ 6.5% [strict]; > 6.5 to ≤ 7.0% [intermediate]; > 7.0 to ≤ 7.5% [lenient]).

**Results:**

8568 patients with T2DM and hypertension were enrolled. 6691 (78.1%) had 12-month follow-up. Patients who were assigned a strict HbA1c treatment target (n = 2644) were younger, had shorter diabetes duration, and less comorbidity in comparison to those with intermediate (n = 2912) or lenient targets (n = 1135). Only 53.1% of patients achieved their HbA1c treatment target (46.2% [strict], 56.8% [intermediate], 59.4% [lenient]). There was little sign of treatment intensification for patients that had not achieved their HbA1c target.

**Conclusions:**

Achievement of treatment targets was poor, leaving many patients with sub-optimal blood glucose levels. The apparent reluctance of physicians to intensify antidiabetic drug therapy is alarming, especially considering the evidence pointing to an association of hyperglycaemia and microvascular complications in patients with T2DM.

**Electronic supplementary material:**

The online version of this article (10.1186/s12933-018-0661-8) contains supplementary material, which is available to authorized users.

## Background

Patients with type 2 diabetes mellitus (T2DM) are known to be at higher cardiovascular risk than those without the condition. This risk is increased even further in the presence of hypertension [1]; a comorbidity found in approximately 70–80% of such patients [2, 3]. In order to reduce the risk of cardiovascular complications, current guidelines strongly recommend reducing HbA1c levels to < 7% [4–6], and have evolved over recent years to place emphasis on achieving this via personalised treatment strategies based on individual patient characteristics [7, 8].

The specific HbA1c target should now take into account factors such as age, comorbidity, and diabetes duration. In a recent position statement, the American Diabetes Association (ADA) in conjunction with the European Association for the Study of Diabetes (EASD) suggested that a stricter target, such as HbA1c ≤ 6.5%, may be more appropriate for younger patients, those with a long life expectancy, those with shorter disease duration, and those with no significant cardiovascular disease [9, 10]. Conversely, a more lenient level, such as < 8%, may be adequate for older patients, those with a shorter life expectancy, those with long disease duration, those with a history of hypoglycaemia, and those with advanced cardiovascular disease. How these guidelines are being put into practice in the real world, however, is not known.

The DIALOGUE registry was established in order to evaluate differences in treatment strategies for patients with T2DM and comorbid hypertension, and to elucidate what factors influence the setting of individual targets in clinical practice [11]. In previous registry analysis, it was found that patients who had been set stricter target HbA1c levels were younger and less comorbid, while those with more lenient targets had a longer diabetes duration and were more likely to have heart failure (HF), peripheral artery disease (PAD), or neuropathy [12]. Importantly, at the 6-month follow-up point, the achievement of HbA1c targets was generally poor. This indicates that medical treatment at that time was inadequate and required subsequent revision and modification by the treating physician.

The primary aim of the current study was to determine the rate of treatment target achievement at 12 months after baseline so as to evaluate “clinical inertia” (e.g. the reluctance of physicians to respond to missed treatment targets at 6 months by adjusting medication accordingly). We also aimed to identify the variables associated with reaching target HbA1c levels at 12 months, and assess the ways in which treatment was adjusted to achieve it.

## Methods

### Study design

DIALOGUE was a prospective, observational, non‐interventional, disease registry with a follow-up of 12 months, performed across multiple centres in Germany. Diabetologists and primary care physicians were responsible for patient enrolment at centres selected in order to provide a representation of ambulatory care for patients with comorbid diabetes and hypertension. The study protocol, as well as primary and secondary objectives of DIALOGUE, have been previously published in detail [11, 12]. DIALOGUE was registered in the database of the Verband forschender Arzneimittelhersteller (http://www.vfa.de/de/arzneimittel-forschung/datenbanken-zu-arzneimitteln/nisdb).

For the purposes of this particular analysis, baseline enrolment was defined as the point at which the treating physician considered blood glucose lowering medication as inadequate and/or not safe/tolerable and chose to add a further drug or switch treatment to achieve glycaemic control. Decisions regarding individual therapies and HbA1c treatment goals were made solely by the attending physician based on their clinical assessment.

This registry was conducted in accordance with the Declaration of Helsinki, and adhered to the principles of Good Epidemiology Practice. Furthermore, the investigation followed all applicable regulatory requirements, and the study protocol was approved by the ethics committee of the Ruhr University (Bochum, Germany). In addition, all patients provided written informed consent, and DIALOGUE was registered in the database of the Verband forschender Arzneimittelhersteller (http://www.vfa.de/de/arzneimittel-forschung/datenbanken-zu-arzneimitteln/nisdb).

### Patients

Patients were consecutively enrolled based on the following criteria: age ≥ 18 years; T2DM with manifested comorbid hypertension; use of oral mono‐ or dual combination antidiabetic therapy (excluding glucagon-like peptide [GLP-1] analogues and insulin) for the period leading up to enrolment; blood glucose-lowering medication considered inadequate and/or not safe/tolerable by the treating physician; additional oral drug added or drug treatment switched by the treating physician to achieve glycaemic control. Patients were excluded based on the following criteria: current participation in a RCT; not under regular supervision of the treating physician during the study; treated with aliskiren in a dual renin–angiotensin–aldosterone system (RAAS) blockade; pregnancy; diabetes secondary to malnutrition, infection, or surgery; maturity onset diabetes of the young; and known cancer.

### Data collection and quality assurance

Data were entered into a web-based electronic case report form (eCRF). Among other information, the following details were collected: patient characteristics (demographics, medical history, and comorbidities); pharmacological therapy for secondary prevention of cardiovascular complications; glucose profile (fasting glucose, post‐prandial glucose, HbA1c); blood pressure; and body mass index (BMI). At the follow-up points, treatment target attainment was determined by HbA1c level achieved, and the antidiabetic medication being used at the time was recorded. Data quality was validated upon eCRF entry, prior to creation of the analysis data set, and through on-site monitoring (2% of the sites randomly selected).

### Statistical analyses

Continuous variables were summarised using standard descriptive statistics (i.e., mean ± standard deviation, median including interquartile range [IQR]), whereas percentages were calculated for categorical data. Comparisons between treatment groups were performed using Pearson’s Chi squared test for categorical variables and the Kruskal–Wallis test for continuous measures. Predictors for target group selection were identified through multivariate logistic regression analysis, with odds ratios (ORs) and 95% confidence intervals (95% CIs) calculated. All statistical analyses were performed using SAS (release 9.2 or higher; Cary, NC, USA). P values ≤ 0.05 were considered to be significant.

## Results

### Patient flow

**Fig. 1:**
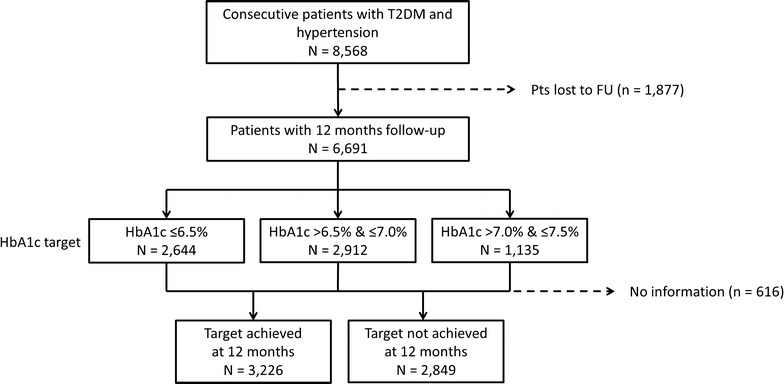
Patient flow. *T2DM* type-2 diabetes, *FU* follow-up

A total of 8568 patients with T2DM and hypertension were enrolled in DIALOGUE, of which 6691 (78.1%) had a follow-up visit at 12 months and comprised the current analysis population. Patients were assigned to one of three groups depending on the target HbA1c level set by their physician (≤ 6.5% [strict]; > 6.5 to ≤ 7.0% [intermediate]; > 7.0 to ≤ 7.5% [lenient]). At baseline, 2644 patients had a strict target, 2912 had an intermediate target, and 1135 had a lenient target (Fig. 1). A total of 6075 patients (90.1% of the current study population) had available information regarding target achievement at 12-month follow-up. The characteristics of the patients lost to follow-up can be found in Additional file 1.

### Patient baseline characteristics

The mean age of the different HbA1c target groups varied, with patients in the strict group having a lower mean age compared to the intermediate and lenient groups (p < 0.0001; Table 1). This also corresponded to a shorter diabetes duration in the strict group compared to the intermediate and lenient group (p < 0.0001). No significant differences in gender or bodyweight were found.

**Table 1:** Patient baseline characteristics by HbA1c treatment target

HbA1c target	≤ 6.5% N = 2644	> 6.5 to ≤ 7.0% N = 2912	> 7.0 to ≤ 7.5% N = 1135	p value
Age (years)	63.3 ± 11.7	66.2 ± 10.5	66.3 ± 10.9	< 0.0001
Female gender (%)	46.3	45.7	44.2	0.51
Diabetes duration (years)	6.1 ± 5.3	7.4 ± 5.8	7.8 ± 6.0	< 0.0001
Bodyweight (kg)	90.2 ± 18.4	90.3 ± 18.4	91.0 ± 18.9	0.45
Any vascular disease (%)^a^	31.3	33.4	36.5	< 0.05
Any diabetes related disease (%)^b^	73.1	80.1	77.4	< 0.0001
Other concomitant disease (%)	41.8	47.1	50.8	< 0.0001
All BG values available (< 6 weeks)^c^	42.9	48.0	38.0	< 0.0001
Metformin (%)	79.9	81.7	81.2	0.23
Sulfonylurea (%)	15.1	19.1	22.5	< 0.0001
Glucosidase inhibitors (%)	1.0	1.5	1.0	0.18
Glinides (%)	2.4	4.0	5.7	< 0.0001
Glitazones (%)	0.5	0.7	0.3	0.18
DPP4-inhibitor (%)	60.0	66.2	63.9	< 0.0001
GLP1-analogue (%)	4.2	5.8	4.4	< 0.05
SGLT-2-inhibitor (%)	1.7	1.9	2.1	0.68
Any insulin (%)	10.9	18.8	22.2	< 0.0001
≥ 3 oral antidiabetic drugs (%)	7.1	11.8	14.0	< 0.0001
≥ 3 antihypertensive drugs (%)	34.4	37.7	37.2	< 0.05
Any non-severe hypoglycaemia (%)^d^	4.6	5.9	6.6	< 0.05
Any severe hypoglycaemia (%)^e^	0.9	0.6	0.3	0.05
PR body weight increase (%)	32.6	35.9	34.7	< 0.01
PR signs of hypoglycaemia (%)	14.9	12.8	14.7	0.66
Mean EQ-5D (mean ± SD)	0.90 ± 0.16	0.88 ± 0.17	0.86 ± 0.18	< 0.0001
Problems with mobility (%)	20.7	28.3	33.0	< 0.0001
Problems with self-care (%)	5.9	10.7	12.9	< 0.0001
Problems with daily activities (%)	15.3	25.4	28.8	< 0.0001
Any pain (%)	41.6	51.4	56.8	< 0.0001
Any anxiety/depression (%)	75.9	72.6	72.2	< 0.01

*HbA1c* glycated haemoglobin, *BG* blood glucose, *DPP* dipeptidyl peptidase, *GLP* glucagon-like peptide, *SGLT* sodium-glucose transporter protein, *PR* patient-reported, *EQ-5D* EuroQol 5D questionnaire regarding health-related quality of life

^a^Any of CAD, prior MI, prior PCI, prior CABG, prior stroke, prior diagnosis of HF

^b^Any of neuropathy, retinopathy, laser coagulation, macular oedema, eye doctor visit, blindness, dialysis, or amputation

^c^Fasting blood glucose, postprandial blood glucose, and HbA1c available

^d^Without symptoms, symptoms but without help, with help—but not medical help or hospitalisation

^e^Symptoms with need for medical help or hospital admission

Approximately a third of patients had vascular disease (strict: 31.3%; intermediate: 33.4%; lenient: 36.5%; p < 0.05), while diabetes-related diseases were more common in the intermediate and lenient groups (73.1, 80.1, and 77.4%, respectively; p < 0.0001).

The proportions of patients receiving ≥ 3 oral antidiabetic drugs at baseline (after addition or switching of medication) was lowest in the strict, followed by the intermediate, and the lenient group (p < 0.0001). The majority of patients were treated with metformin (80.9%) or dipeptidyl peptidase-4 (DPP-4) inhibitors (63.3%), fostered by the study design. Sulfonylureas, glinides and insulin, were all more common in the patient group with a lenient treatment target, while GLP1-A and DPP-4 inhibitors were more common in the intermediate group. On the other hand, similar proportions of patients in each group were being treated with metformin, glucosidase inhibitors, glitazones, or sodium glucose transporter protein 2 (SGLT-2) inhibitors.

Health-related quality of life, as determined using the EQ-5D questionnaire, was reported to be poorer for the patients with the lenient HbA1c target, while those with the strict target reported the highest quality of life (p < 0.0001). The same trend was seen in four out of five categories of the questionnaire (mobility, self-care, daily activities, and pain), while the inverse was true for the fifth category (anxiety).

### HbA1c treatment target achievement at 12 month follow-up

To illustrate treatment target achievement more clearly, we divided patients into 3 tertiles based on their baseline HbA1c values (1st tertile: ≤ 7.0%, 2nd tertile: 7.1–7.9%, 3rd tertile: ≥ 8.0%). This resulted in 2067 patients (35%) in the 1st tertile, 1793 patients (30%) in the 2nd tertile, and 2032 patients (35%) in the 3rd tertile. Physicians tended to select a treatment target close to the patient’s baseline HbA1c value, excepting patients in the 3rd tertile for whom a lenient treatment target was assigned less often than an intermediate target: Of the patients in the 1st tertile the majority were assigned a strict treatment target of ≤ 6.5% (1310 patients, 63.4%). Of the patients in the 2nd tertile, the majority were assigned an intermediate target of > 6.5 and ≤ 7.0% (931 patients, 51.9%), and of the patients in the 3rd tertile, the majority were also assigned an intermediate target of > 6.5 and ≤ 7.0% (1006 patients, 49.5%).

**Fig. 2:**
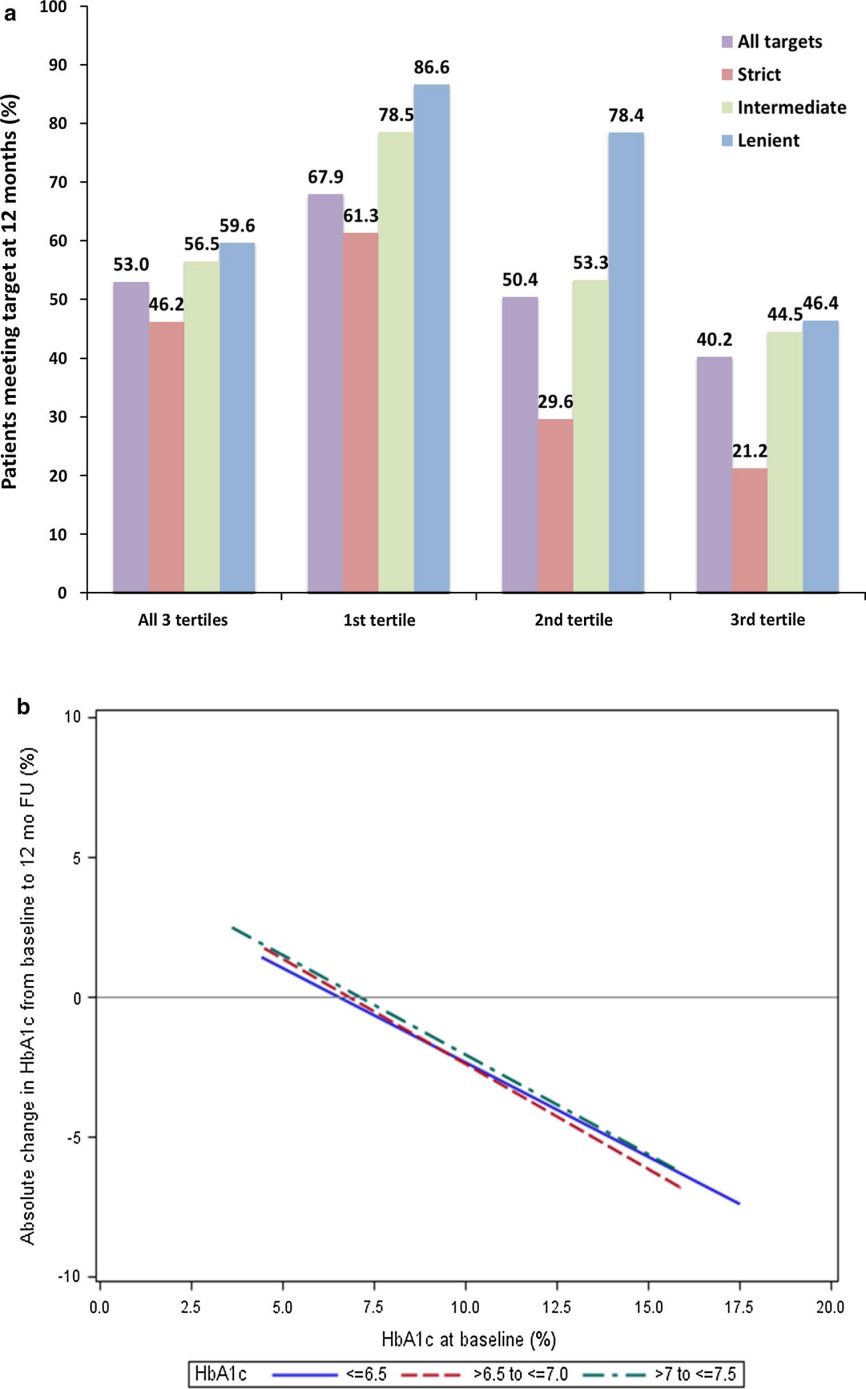
**a** Proportions of patients meeting treatment targets at 12 months, stratified by baseline HbA1c tertile and target type. **b** Change in HbA1c at 12-month follow-up by baseline HbA1c and treatment target group. Only patients with valid baseline and 12 month follow-ups including complete information on HbA1c levels were included. Percentages refer to the proportion of patients within the specific subgroup represented by each column

Overall, a total of 53.0% of patients achieved a HbA1c level within, or below their treatment target at 12 months (Fig. 2a). When grouped by baseline tertiles, this was true for 67.9, 50.4, and 40.2% of patients in the 1st, 2nd and 3rd tertiles, respectively. When grouped by target type, the proportion of patients meeting their targets was lowest in the strict, higher in the intermediate, and the highest in the lenient group; a trend that was also seen within each tertile. However, the magnitude of the proportion of patients meeting their targets generally decreased through tertile 1–3. Accordingly, the highest proportion of patients reaching their target (86.6%) was recorded for patients in the 1st tertile with a lenient target, while conversely, the lowest proportion (21.2%) was seen for patients in the 3rd tertile with a strict target.

### Change in HbA1c at 12 month follow-up

There was a linear association between baseline HbA1c levels and absolute reduction in HbA1c at 12 months, where a higher baseline HbA1c value correlated with a greater absolute reduction, regardless of assigned treatment target (Fig. 2b).

**Fig. 3:**
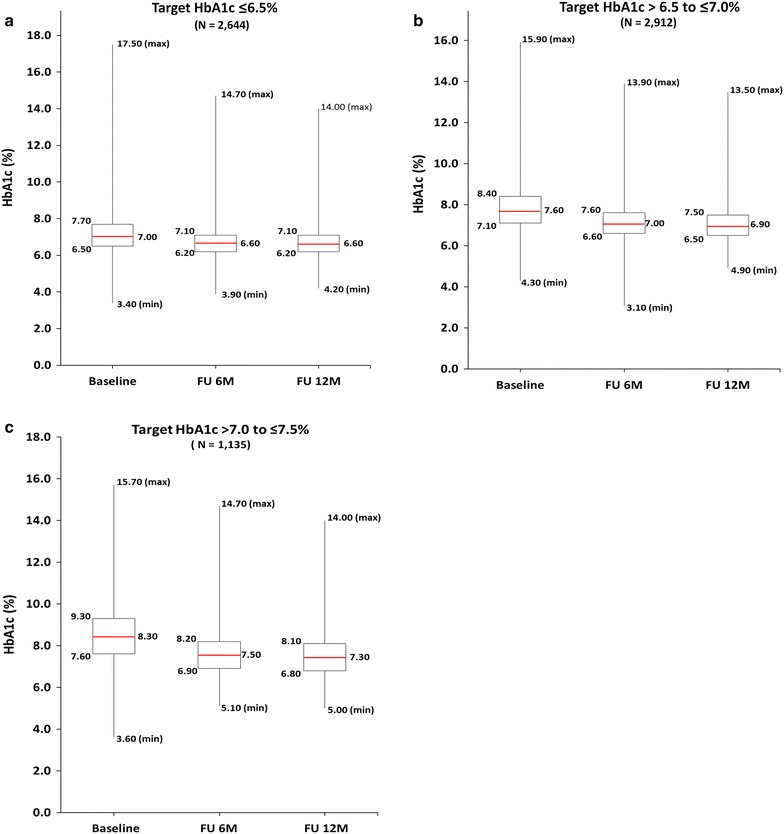
HbA1c reduction by treatment target (Baseline, 6 and 12 months). **a** Patients with HbA1c target ≤6.5%. **b** Patients with HbA1c target > 6.5 to ≤7.0%. **c** Patients with HbA1c target > 7.0 to ≤7.5%.  Data presented as median, 25th and 75th percentiles, maximum, and minimum values

As shown in Fig. 3, the median HbA1c value at baseline for the patients with a treatment target of ≤ 6.5% was 7.00%. This decreased to 6.60% at 12 months, giving a mean reduction from baseline of 0.50 ± 1.19% (p < 0.0001). For the patients with a treatment target of > 6.5 to ≤ 7.0%, the median HbA1c value at baseline was 7.60%. This decreased to 6.90% at 12 months, giving a mean reduction from baseline of 0.81 ± 1.24% (p < 0.0001). For the patients with a treatment target of > 7.0 to ≤ 7.5%, the median HbA1c value at baseline was 8.30%. This decreased to 7.30% at 12 months, giving a mean reduction from baseline of 1.07 ± 1.49% (p < 0.0001).

### Predictors of treatment target achievement

After multivariate analysis, certain factors were found to be predictive of treatment target achievement (Table 2; Additional file 2). Overall, patients with an age above the median (OR: 1.25), those with all blood glucose values available (OR: 1.29), and those with all renal values available (OR: 1.24) were more likely to achieve their treatment target. Compared to patients with a strict HbA1c target, those for whom this target was intermediate (OR: 5.31) or lenient (OR: 3.02) were more likely to achieve their treatment target.

**Table 2:** Multivariable predictors of treatment target achievement at 12 months

	Multivariable OR (95% CI)
Age > median	
Yes vs. no	*1.25 (1.07*–*1.45)*
Female gender	
Yes vs. no	1.03 (0.91–1.17)
Diabetes duration > median	
Yes vs. no	*0.65 (0.58–0.73)*
Bodyweight > median	
Yes vs. no	0.91 (0.80–1.02)
HbA1c treatment target	
> 7.0% vs. ≤ 6.5%	*3.02 (2.61–3.49)*
> 6.5 to ≤ 7.0% vs. ≤ 6.5%	*5.31 (4.38–6.44)*
HbA1c baseline tertile	
3rd tertile vs. 1st tertile	*0.25 (0.22–0.30)*
2nd tertile vs. 1st tertile	*0.15 (0.13–0.18)*
Care-dependent	
Yes vs. no	0.84 (0.57–1.24)
Not working	
Yes vs. no	0.97 (0.83–1.14)
< 9 years of school education	
Yes vs. no	*0.86 (0.76–0.98)*
Patient lives alone	
Yes vs. no	0.88 (0.77–1.02)
< 1 h per week of physical activity	
Yes vs. no	0.93 (0.82–1.06)
Any vascular disease^a^	
Yes vs. no	0.96 (0.84–1.09)
Any diabetes related disease^b^	
Yes vs. no	1.04 (0.90–1.20)
Other concomitant disease	
Yes vs. no	0.94 (0.84–1.06)
All lipid values available (< 6 weeks)^c^	
Yes vs. no	1.03 (0.90–1.18)
All BG values available (< 6 weeks)^d^	
Yes vs. no	*1.29 (1.14–1.45)*
All renal lab values available^e^	
Yes vs. no	*1.24 (1.09–1.41)*
≥ 3 oral antidiabetic drugs at BL	
Yes vs. no	*0.59 (0.49–0.72)*
People receiving any insulin	
Yes vs. no	*0.82 (0.70–0.97)*
≥3 antihypertensive drugs at BL	
Yes vs. no	1.07 (0.95–1.21)
Any non-severe hypoglycaemia^f^	
Yes vs. no	0.95 (0.74–1.23)
Any severe hypoglycaemia^g^	
Yes vs. no	0.53 (0.25–1.13)

*HbA1c* glycated haemoglobin, *BG* blood glucose

^a^Any of CAD, prior MI, prior PCI, prior CABG, prior stroke, prior diagnosis of HF

^b^Any of neuropathy, retinopathy, laser coagulation, macular oedema, eye doctor visit, blindness, dialysis, or amputation

^c^Total cholesterol, HDL-C, LDL-C, and TG available

^d^Fasting blood glucose, postprandial blood glucose, and HbA1c available

^e^Serum creatinine and information on macroalbuminuria available

^f^Without symptoms, symptoms but without help, with help—but not medical help or hospitalisation

^g^Symptoms with need for medical help or hospital admission. Patient numbers and univariate odds ratios are available in Additional file 2

Conversely, patients with a diabetes duration above the median (OR: 0.65), those with less than 9 years of school education (OR: 0.86), those being treated with ≥ 3 oral antidiabetic drugs at baseline (OR: 0.59), and those being treated with insulin (OR: 0.82) were less likely to achieve their treatment target. Compared to patients in the 1st tertile for HbA1c level at baseline, those in the 2nd (OR: 0.15) and 3rd (OR: 0.25) tertiles were less likely to achieve their treatment target.

### Treatment changes by target attainment at 12 months

For the group of patients who had not achieved their treatment target at the 6-month follow-up, there were few changes in the use of antidiabetic therapy from baseline to 6 months or from 6 months to 12 months (Table 3). The proportions of patients being treated with each of the drugs did not change greatly over time, although insulin use increased slightly at each time point. There were also only small differences between patients that did and did not achieve their treatment target at 12 months. Insulin use at 12 months was slightly higher for the patients that did not achieve their target (29.9%) than for those that did (24.6%), as was SGLT-2 inhibitor use (4.3% vs. 2.2%) and DPP4 inhibitor use (64.8% vs. 62.3%).

**Table 3:** Changes in treatment for patients not at target at 6 months by target achievement at 12 months

	Target achieved at 12 months (N = 763)		Target not achieved at 12 months (N = 2136)	
	6 months % (Δ from BL)	12 months % (Δ from 6 mo)	6 months % (Δ from BL)	12 months % (Δ from 6 mo)
Metformin	82.3 (− 0.9)	82.3 (–)	82.6 (−0.6)	81.7 (−0.9)
Sulfonylurea	20.8 (+ 0.2)	20.1 (− 0.7)	20.9 (+ 0.3)	20.7 (− 0.2)
Glucosidase inhibitor	1.6 (+ 1.4)	1.3 (− 0.3)	1.0 (− 0.2)	0.9 (− 0.1)
Glinide	3.5 (− 0.6)	3.5 (–)	4.6 (+ 0.5)	5.0 (+ 0.4)
Glitazone	0.7 (+ 0.3)	0.8 (+ 0.1)	0.2 (− 0.2)	0.3 (+ 0.1)
DPP-4 inhibitor	63.0 (− 2.8)	62.3 (− 0.7)	66.7 (+ 0.9)	64.8 (− 1.9)
GLP-1 analogue	6.9 (+ 1.1)	6.6 (− 0.3)	6.1 (+ 0.3)	5.8 (− 0.3)
SGLT-2 inhibitor	1.7 (− 1.0)	2.2 (+ 0.5)	3.9 (+ 1.2)	4.3 (+ 0.4)
Any insulin	21.2 (+ 1.8)	24.6 (+ 3.4)	25.0 (+ 5.6)	29.9 (+ 4.9)

*BL* baseline, *DPP* dipeptidyl peptidase, *GLP* glucagon-like peptide, *SGLT* sodium-glucose transporter protein

## Discussion

The targets assigned to the patients in DIALOGUE were generally in line with those recommended in recent guidelines. However, the rate of target achievement was fairly poor across the board, particularly in patients with higher baseline HbA1c values. Despite this, a relatively small degree of treatment intensification was apparent, suggesting substantial clinical inertia.

### Treatment target achievement

The achievement of HbA1c targets in patients with diabetes has been previously shown to be poor [13–15]. This is of particular concern for patients with additional risk factors, such as hypertension or cardiovascular disease, who are at increased risk of adverse events. In the EUROASPIRE IV study, low proportions of high-risk diabetic patients both with and without established cardiovascular disease had achieved their target HbA1c level [16, 17].

The present data, and that previously reported for the 6-month follow-up of DIALOGUE [12], show that patients who were assigned a strict HbA1c treatment target were younger, had shorter diabetes duration, less comorbidity, and considered themselves to have a better quality of life in comparison to the other groups. The allocation of a strict target for these patients is in agreement with the position statement from the ADA/EASD, where a lower HbA1c goal was said to be appropriate for patients with these same characteristics [6, 9, 10]. However, only 46.2% of this patient group achieved their treatment target at 12 months, fewer than for each of the other two groups. In addition, they displayed the smallest mean change in HbA1c from baseline to 12-month follow-up. Over half were in the 1st baseline HbA1c tertile, indicating that they had lower HbA1c levels to start with and therefore did not require a large reduction in order to achieve their target. The magnitude of HbA1c reduction (− 0.50%) and the percentage target achievement (46.2%) at 12 months were almost identical to those found at the 6-month follow up point (− 0.40 and 46.3%, respectively) [12]. This suggests that there was very little change in blood glucose level during the period from 6 to 12 months.

The patients that were assigned a lenient treatment target had longer diabetes duration and a higher prevalence of vascular disease, again in agreement with guidelines [6, 9, 10]. Goal achievement was low at 59.6%, although it was higher than that found for the patients with a strict target. This is in agreement with the multivariable analysis, which showed that having a lenient target vs. a strict target was strongly predictive of goal achievement. Over half of the patients in the lenient treatment target group were in the 3rd baseline HbA1c tertile, indicating higher blood glucose levels and a more urgent need for drastic HbA1c management to reduce cardiovascular risk. Accordingly, the mean reduction in HbA1c was largest for this group of patients (and in fact any patients with high initial HbA1c levels, regardless of treatment targets). The data show that lenient target patients were receiving more intensive antidiabetic therapy at baseline, with a higher proportion of them being treated with insulin or sulfonylurea, and more of them taking ≥ 3 oral antidiabetic drugs. This may account for the greater decrease in HbA1c levels; however, it is clear that the reduction was inadequate for a large number of patients. Another finding that contrasts with that for the strict target group is that the median HbA1c level not only decreased from baseline to 6 months, but also from 6 months to 12 months. Target achievement was also seen to improve during this time, from 52.2% at 6 months to 59.6% at 12 months [12]. These improvements suggest that the antidiabetic treatment regimens were having some effect; though the meagre 0.13% decrease in median HbA1c level from 6 to 12 months shows that it was at best modest and by no means optimal.

The intermediate target HbA1c of < 6.5 to ≤ 7.0% is that which most closely corresponds to the value advocated in current guidelines for reducing the risk of cardiovascular events in patients with T2DM [5, 18]. In the present study, patients in this group were of a similar age to those of the lenient target group, while values for diabetes duration and the presence of vascular disease were between those of the strict and lenient patients. This shows that, in the absence of extenuating factors, these patients were assigned the generally accepted HbA1c target. The rate of treatment target achievement at 12 months for all patients assigned an intermediate target fell between those of the strict and lenient groups (56.5%), and was slightly higher than that found at the 6-month follow-up (50.2%) [12]. Having an intermediate vs. a strict target was also found by multivariable analysis to be predictive of goal achievement.

There were a number of factors that were predictive of a failure to achieve the particular treatment target assigned by the physician. Patients with diabetes duration above the median value, < 9 years of school education, and those being treated with insulin or ≥ 3 oral antidiabetic drugs at baseline were found to be less likely to reach their assigned HbA1c level. Most of these factors correspond to more severe T2DM, and could suggest that a more lenient HbA1c target may have been appropriate for a proportion of patients in the intermediate group. Patients in the 2nd and 3rd tertiles were also less likely to achieve their target than those in the 1st tertile, further suggesting that target setting may have been particularly too stringent in these groups of patients.

Patients for whom all blood glucose and renal laboratory values from the most recent 6 weeks were available were more likely to achieve their target. This finding suggests that these patients had greater contact with their physician, resulting in improved glycaemic control. The finding that patients aged above the mean value of the study population were also more likely to reach their target may have been due to a greater conscientiousness in terms of treatment adherence and lifestyle modification in these patients.

### Treatment adjustment from baseline to follow-up

In general, the proportion of patients receiving each antidiabetic drug were lower for those that achieved their treatment target at 12 months than for those that did not. This pattern was found at both 6 and 12 months, with little variation in treatment during follow up period. This indicates that HbA1c levels were more adequately controlled in these patients than in those that did not achieve their target. It may be expected that a patient not achieving their assigned HbA1c target may have their antidiabetic medication changed by their physician; however, in the present study, this appears not to be the case. Despite the high number of patients not reaching their treatment target at 6 months, antidiabetic therapy did not change considerably up to the 12-month follow-up point. The only alteration of note is the increase in insulin use between baseline and 6 months and between 6 months and 12 months. This finding indicates a degree of reluctance on the part of the physician to intensify treatment, even when faced with evidence of inadequate glycaemic control. Such clinical inertia has been previously reported in the setting of T2DM [19–22]. Factors such as a lack of knowledge of recent guidelines, poor clinical judgement, and response to patients’ attitudes regarding drug treatment may all contribute to this unfortunate state of affairs [23, 24]. An additional reason is uncertainty over what the most appropriate treatment strategy for an individual patient would be, with many antidiabetic drugs now available. This was recently highlighted by Ampudia-Blasco et al. who in response to the aforementioned ADA/EASD recommendations, provided a decision support tool for use by physicians when prescribing antidiabetic therapy [25].

### Limitations

One limitation of the present analysis is the presence of comorbid hypertension, which may limit the applicability of the data to all T2DM patients. However, as around 70–80% of individuals with T2DM also have hypertension, our data are highly representative of the vast majority of patients in the real-world situation. It should also be noted that the study was performed in a single country; therefore, the data may not be generalizable to the global T2DM population. A further limitation is that the follow-up duration presented here was only 12 months. Ongoing monitoring of the same patients will allow for target achievement after further alterations in treatment to be evaluated. A final issue is that any changes made to treatment targets during the 12-month follow-up were not taken into account. It is possible that factors such as hypoglycaemia occurrence or cardiovascular events may have altered the treatment strategy initiated by the physician.

## Conclusions

Treatment targets set by physicians were generally in line with the recommendations set out in the ADA/EASD position statement. However, achievement of each of these targets was poor, leaving many patients with sub-optimal blood glucose levels. The apparent reluctance of physicians to intensify antidiabetic drug therapy is worrying, especially considering the large body of evidence pointing to an association of hyperglycaemia and microvascular complications in patients with T2DM.

## Additional files

**Additional file 1.** Patient characteristics of total population. Details the characteristics of patients with and without follow-up information, along with statistical comparison of differences.

**Additional file 2.** Full univariate and multivariate regression data set. Contains patient numbers, univariate odds ratios and multivariate odds ratios for predictors of target achievement at 12 months.

## Abbreviations

ADA: American Diabetes Association
BG: blood glucose
BMI: body mass index
DDP-4: dipeptidyl peptidase-4
EASD: European Association for the Study of Diabetes
eCRF: electronic case report form
GLP‐1: glucagon-like peptide-1
HbA1c: glycated haemoglobin
PAD: peripheral artery disease
PR: patient reported
SGLT: sodium glucose transporter protein
T2DM: type-2 diabetes mellitus
