# Supplementary material for: Individualised treatment targets in patients with type-2 diabetes and hypertension
Source: Cardiovasc Diabetol. 2018 Jan 22;17:18. doi: 10.1186/s12933-018-0661-8 (PMC5778654; doi:10.1186/s12933-018-0661-8)
Supplement: Supplementary file 1 — Additional file 1. Patient characteristics of total population. Details the characteristics of patients with and without follow-up information, along with statistical comparison of differences. [file 12933_2018_661_MOESM1_ESM.docx]

**Additional Table 1: Patient characteristics of total population**

|  | All patients included | | | All patients with a 12 months FU | | | |
| --- | --- | --- | --- | --- | --- | --- | --- |
|  | Pts with 12 mo FU | Pts lost to FU | p-value | HbA1c ≤6.5% | HbA1c >6.5 to ≤7.0% | HbA1c >7.0 to ≤7.5% | p-value |
| Patients (n) | 6691 | 1877 |  | 2644 | 2912 | 1135 |  |
| Age (years) | 65.1 ± 11.1 | 65.1 ± 12.0 | 0.70 | 63.3 ± 11.7 | 66.2 ± 10.5 | 66.3 ± 10.9 | <0.0001 |
| Female gender (%) | 45.7 | 44.8 | 0.48 | 46.3 | 45.7 | 44.2 | 0.51 |
| Diabetes duration (years) | 7.0 ± 5.7 | 6.7 ± 5.4 | 0.07 | 6.1 ± 5.3 | 7.4 ± 5.8 | 7.8 ± 6.0 | <0.0001 |
| Bodyweight (kg) | 90.4 ± 18.5 | 90.2 ± 18.6 | 0.75 | 90.2 ± 18.4 | 90.3 ± 18.4 | 91.0 ± 18.9 | 0.45 |
| Care-dependent (%) | 2.3 | 3.7 | <0.01 | 1.4 | 2.4 | 4.3 | <0.0001 |
| Not working (%) | 64.5 | 62.9 | 0.23 | 58.9 | 68.8 | 66.2 | <0.0001 |
| <9 years of school education (%) | 32.4 | 27.5 | <0.0001 | 31.3 | 31.7 | 39.3 | <0.0001 |
| Patient lives alone (%) | 20.2 | 21.5 | 0.24 | 18.0 | 21.0 | 22.9 | <0.001 |
| <1h per week of physical activity (%) | 32.8 | 36.1 | <0.05 | 31.3 | 31.7 | 39.3 | <0.0001 |
| Any vascular disease^1^ | 32.9 | 31.1 | 0.15 | 31.3 | 33.4 | 36.5 | <0.05 |
| Any diabetes related disease^2^ | 76.9 | 73.2 | <0.01 | 73.1 | 80.1 | 77.4 | <0.0001 |
| Other concomitant disease | 45.6 | 47.8 | 0.11 | 41.8 | 47.1 | 50.8 | <0.0001 |
| All lipid values available (<6 weeks)^3^ | 68.0 | 55.7 | <0.0001 | 67.2 | 68.1 | 69.8 | 0.29 |
| All BG values available (<6 weeks)^4^ | 44.3 | 37.4 | <0.0001 | 42.9 | 48.0 | 38.0 | <0.0001 |
| All renal lab values available^5^ | 61.8 | 49.5 | <0.0001 | 59.4 | 64.3 | 60.9 | <0.001 |
| Metformin (%) | 80.9 | 76.2 | <0.0001 | 79.9 | 81.7 | 81.2 | 0.23 |
| Sulfonylurea (%) | 18.1 | 15.2 | <0.01 | 15.1 | 19.1 | 22.5 | <0.0001 |
| Glucosidase inhibitors (%) | 1.2 | 0.5 | <0.05 | 1.0 | 1.5 | 1.0 | 0.18 |
| Glinides (%) | 3.6 | 2.8 | 0.09 | 2.4 | 4.0 | 5.7 | <0.0001 |
| Glitazones (%) | 0.6 | 0.4 | 0.33 | 0.5 | 0.7 | 0.3 | 0.18 |
| DPP4-I (%) | 63.3 | 59.0 | <0.01 | 60.0 | 66.2 | 63.9 | <0.0001 |
| GLP1-A (%) | 4.9 | 4.4 | 0.40 | 4.2 | 5.8 | 4.4 | <0.05 |
| SGLT-2-I (%) | 1.9 | 1.4 | 0.19 | 1.7 | 1.9 | 2.1 | 0.68 |
| Any insulin (%) | 16.2 | 12.2 | <0.0001 | 10.9 | 18.8 | 22.2 | <0.0001 |
| ≥3 oral antidiabetic drugs (%) | 10.3 | 6.0 | <0.0001 | 7.1 | 11.8 | 14.0 | <0.0001 |
| ≥3 antihypertensive drugs (%) | 36.3 | 30.7 | <0.0001 | 34.4 | 37.7 | 37.2 | <0.05 |
| Any non-severe hypoglycaemia^6^ (%) | 5.5 | 7.5 | <0.01 | 4.6 | 5.9 | 6.6 | <0.05 |
| Any severe hypoglycaemia^7^ (%) | 0.7 | 1.1 | <0.05 | 0.9 | 0.6 | 0.3 | 0.05 |
| PR body weight increase (%) | 23.9 | 27.6 | <0.0001 | 32.6 | 35.9 | 34.7 | <0.01 |
| PR signs of hypoglycaemia (%) | 14.1 | 18.2 | <0.0001 | 14.9 | 12.8 | 14.7 | 0.66 |
| Mean EQ-5D (mean ± SD) | 0.88 ± 0.17 | 0.84 ± 0.23 | <0.0001 | 0.90 ± 0.16 | 0.88 ± 0.17 | 0.86 ± 0.18 | <0.0001 |
| Problems with mobility (%) | 26.0 | 29.3 | <0.05 | 20.7 | 28.3 | 33.0 | <0.0001 |
| Problems with self-care (%) | 9.2 | 14.6 | <0.0001 | 5.9 | 10.7 | 12.9 | <0.0001 |
| Problems with daily activities (%) | 22.0 | 29.7 | <0.0001 | 15.3 | 25.4 | 28.8 | <0.0001 |
| Any pain (%) | 48.4 | 56.3 | <0.0001 | 41.6 | 51.4 | 56.8 | <0.0001 |
| Any anxiety/depression (%) | 26.2 | 33.2 | <0.0001 | 75.9 | 72.6 | 72.2 | <0.01 |
