# Supplementary material for: Individualised treatment targets in patients with type-2 diabetes and hypertension
Source: Cardiovasc Diabetol. 2018 Jan 22;17:18. doi: 10.1186/s12933-018-0661-8 (PMC5778654; doi:10.1186/s12933-018-0661-8)
Supplement: Supplementary file 2 — Additional file 2. Full univariate and multivariate regression data set. Contains patient numbers, univariate odds ratios and multivariate odds ratios for predictors of target achievement at 12 months. [file 12933_2018_661_MOESM2_ESM.docx]

**Additional Table 2: Full univariate and multivariate regression data set**

|  |  | Target achieved (%) (n=3226) | Target not achieved (%) (n=2849) | Univariable OR (95%CI) | Multivariable OR (95%CI) |
| --- | --- | --- | --- | --- | --- |
| Age > median | Yes vs. No | 53.6 | 44.4 | 1.44 (1.31-1.60) | 1.25 (1.07-1.45) |
| Female gender | Yes vs. No | 47.4 | 44.9 | 1.10 (1.00-1.22) | 1.03 (0.91-1.17) |
| Diabetes duration > median | Yes vs. No | 44.1 | 53.9 | 0.68 (0.61-0.75) | 0.65 (0.58-0.73) |
| Bodyweight > median | Yes vs. No | 46.7 | 53.0 | 0.78 (0.70-0.86) | 0.91 (0.80-1.02) |
| HbA1c Treatment Target | >7.0% vs. ≤6.5% | 35.7 | 24.6 | 1.53 (1.37-1.71) | 3.02 (2.61-3.49) |
|  | >6.5% to ≤7.0% vs. ≤6.5% | 58.3 | 47.8 | 1.70 (1.47-1.97) | 5.31 (4.38-6.44) |
| HbA1c baseline tertile | 3^rd^ tertile vs. 1^st^ tertile | 38.8 | 69.5 | 0.28 (0.24-0.32) | 0.25 (0.22-0.30) |
|  | 2^nd^ tertile vs. 1^st^ tertile | 44.1 | 65.7 | 0.41 (0.36-0.47) | 0.15 (0.13-0.18) |
| Care-dependent | Yes vs. No | 2.2 | 2.3 | 0.94 (0.67-1.32) | 0.84 (0.57-1.24) |
| Not working | Yes vs. No | 67.0 | 61.5 | 1.27 (1.14-1.41) | 0.97 (0.83-1.14) |
| <9 years of school education | Yes vs. No | 32.7 | 32.7 | 1.00 (0.90-1.11) | 0.86 (0.76-0.98) |
| Patient lives alone | Yes vs. No | 19.9 | 20.9 | 0.94 (0.83-1.06) | 0.88 (0.77-1.02) |
| <1h per week of physical activity | Yes vs. No | 31.4 | 33.6 | 0.90 (0.81-1.01) | 0.93 (0.82-1.06) |
| Any vascular disease^1^ | Yes vs. No | 32.8 | 30.8 | 1.10 (0.99-1.23) | 0.96 (0.84-1.09) |
| Any diabetes related disease^2^ | Yes vs. No | 78.2 | 76.9 | 1.08 (0.96-1.22) | 1.04 (0.90-1.20) |
| Other concomitant disease | Yes vs. No | 45.3 | 45.5 | 0.99 (0.90-1.10) | 0.94 (0.84-1.06) |
| All lipid values avail. (<6 weeks)^3^ | Yes vs. No | 70.6 | 67.3 | 1.17 (1.05-1.30) | 1.03 (0.90-1.18) |
| All BG values available (<6 weeks)^4^ | Yes vs. No | 48.0 | 42.0 | 1.27 (1.15-1.41) | 1.29 (1.14-1.45) |
| All renal lab values available^5^ | Yes vs. No | 66.2 | 60.1 | 1.31 (1.18-1.45) | 1.24 (1.09-1.41) |
| ≥3 oral antidiabetic drugs at BL | Yes vs. No | 7.3 | 13.6 | 0.50 (0.42-0.59) | 0.59 (0.49-0.72) |
| People receiving any insulin | Yes vs. No | 13.0 | 19.9 | 0.60 (0.53-0.69) | 0.82 (0.70-0.97) |
| ≥3 antihypertensive drugs at BL | Yes vs. No | 36.5 | 34.7 | 1.08 (0.97-1.20) | 1.07 (0.95-1.21) |
| Any non-severe hypoglycaemia^6^ | Yes vs. No | 5.5 | 5.4 | 1.02 (0.81-1.28) | 0.95 (0.74-1.23) |
| Any severe hypoglycaemia^7^ | Yes vs. No | 0.5 | 0.9 | 0.62 (0.33-1.16) | 0.53 (0.25-1.13) |

*Legend:* HbA1c, glycated haemoglobin; TT, treatment target; BG, blood glucose. ^1^ Any of CAD, prior MI, prior PCI, prior CABG, prior stroke, prior diagnosis of HF; ^2^ Any of neuropathy, retinopathy, laser coagulation, macular oedema, eye doctor visit, blindness, dialysis, or amputation; ^3^ Total cholesterol, HDL-C, LDL-C, and TG available; ^4^ Fasting blood glucose, postprandial blood glucose, and HbA1c available; ^5^ Serum creatinine & information on macroalbuminuria available; ^6^ Without symptoms, symptoms but without help, with help – but not medical help or hospitalisation; ^7^ Symptoms with need for medical help or hospital admission.
